# Supplementary material for: Temperamental Development among Preterm Born Children. An RCT Follow-up Study
Source: Children (Basel). 2020 Apr 23;7(4):36. doi: 10.3390/children7040036 (PMC7230507; doi:10.3390/children7040036)
Supplement: Supplementary file 1 [file children-07-00036-s001.pdf]

Supplementary Materials:

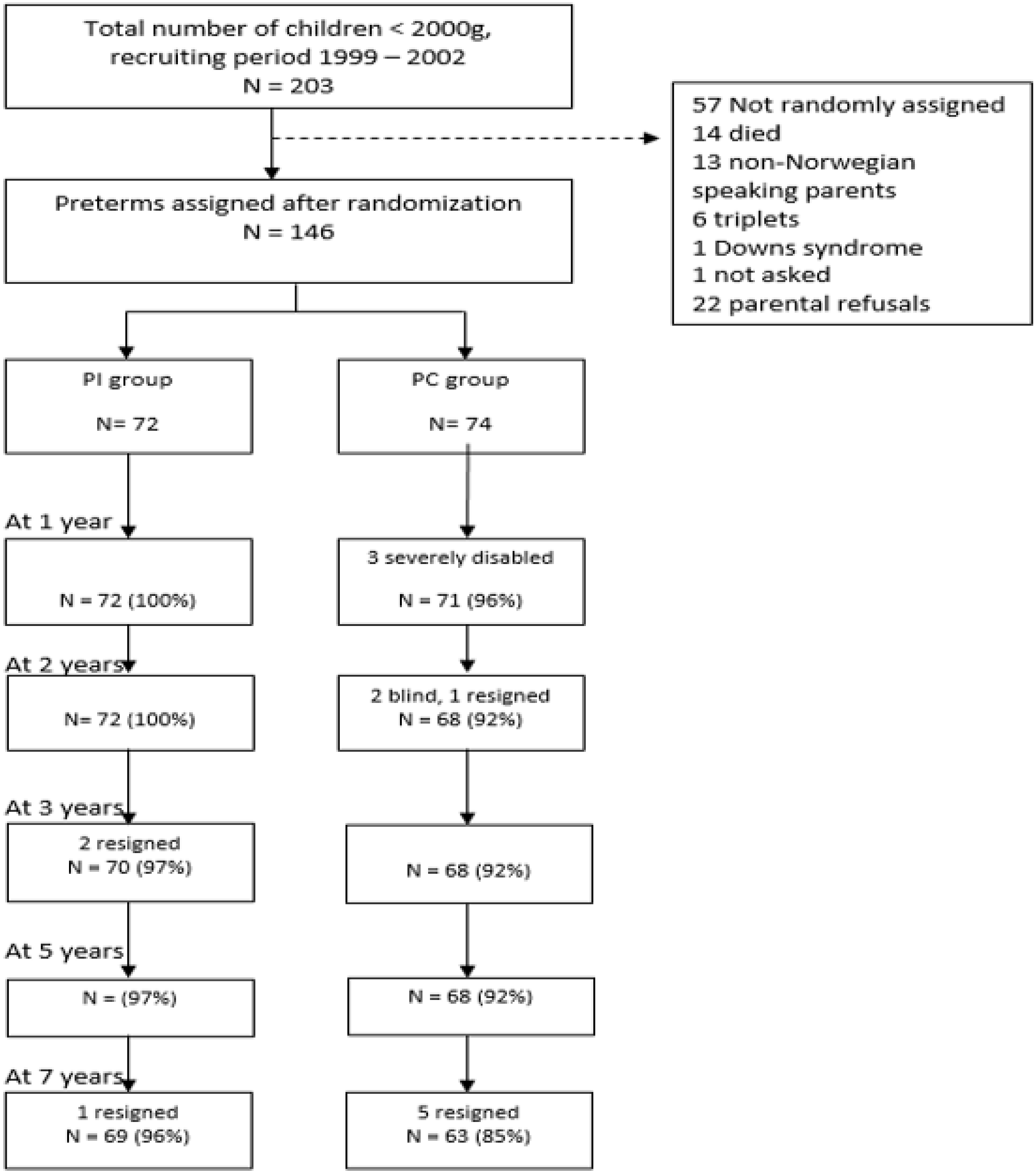

Figure S1. Tromsø Intervention Study on Preterms (TISP). Flow of participants in the two groups with preterm born infants from recruitment until age seven.

Table S1. Fixed effects of time and group-belonging on mothers reports on five temperament dimensions.

	Shyness	Sociality	Emotionality	Activity	Soothability
Time	F(1,115) < 0.1 ns.	F(1,116) = 2.2 ns.	F(1,111) = 5.1 *	F(1,116) = 60.9 ***	F(1,115) = 2.7 ns.
Group	F(1,124) < 0.1 ns.	F(1,119) = 0.6 ns.	F(1,121) = 9.7 **	F(1,120) = 1.8 ns	F(1,120) = 3.7 ns. [2]
Time x group	F(1,115) = 0.4 ns.	F(1,116) = 0.1 ns.	F(1,111) = 1.8 ns.	F(1,116) = 3.6 ns [3]	F(1,115) = 0.5 ns.
Tot. Stress [1]	F(1,125) = 9.2 **	F(1,122) = 7.8 **	F(1,126) = 8.3 **	F(1,128) = 6.3 *	F(1,128) = 25.2 ***
Years educ.	F(1,123) = 1.6 ns.	F(1,119) = 0.4 ns.	F(1,122) = 1.2 ns.	F(1,116) = 0.1 ns.	F(1,121) = 1.1 ns.

ns. $p > 0.05$, * $p < 0.05$, ** $p < 0.01$, *** $p < 0.001$, non-significant results toned down. [1]: PSI, total stress reported by mothers at children's age of 1 year [2]: $p = 0.056$ [3]: $p = 0.06$.

Table S2. Fixed effects of time and group-belonging on fathers reports on five temperament dimensions.

	Shyness	Sociality	Emotionality	Activity	Soothability
Time	F(1,93) < 0.1 ns.	F(1,93) < 0.1 ns.	F(1,94) = 1.3 ns.	F(1,91) = 61.7 ***	F(1,95) = 0.3 ns.
Group	F(1,100) = 0.2 ns.	F(1,102) = 0.5 ns.	F(1,98) = 0.6 ns.	F(1,98) = 0.7 ns.	F(1,100) = 14.2 ***
Time x Group	F(1,94) = 0.4 ns.	F(1,93) < 0.1 ns.	F(1,94) = 4.8 *	F(1,91) = 0.3 ns.	F(1,95) = 1.7 ns.
Total stress [1]	F(1,99) = 7.5 **	F(1,99) = 27.1 ***	F(1,99) = 9.5 **	F(1,98) = 1.9 ns.	F(1,98) = 3.8 ns.
Years educ.	F(1,100) < 0.1 ns.	F(1,98) < 0.1 ns.	F(1,101) = 4.7 *	F(1,99) = 0.7 ns.	F(1,98) = 0.2 ns.

ns. $p > 0.05$, * $p < 0.05$, ** $p < 0.01$, *** $p < 0.001$, non-significant results toned down. [1]: PSI, total stress reported by fathers at children's age of 1 year.
